# The sex-specific metabolic signature of C57BL/6NRj mice during aging

**DOI:** 10.1038/s41598-022-25396-8

**Published:** 2022-12-06

**Authors:** Doruntina Bresilla, Hansjoerg Habisch, Iva Pritišanac, Kim Zarse, Warisara Parichatikanond, Michael Ristow, Tobias Madl, Corina T. Madreiter-Sokolowski

**Affiliations:** 1 Molecular Biology and Biochemistry, Gottfried Schatz Research Center, Medical University of Graz, Neue Stiftingtalstraße 6/VI, 8010 Graz, Austria; 2 Laboratory of Energy Metabolism, Department of Health Sciences and Technology, Institute of Translational Medicine, ETH Zurich, Schorenstrasse 16, 8603 Schwerzenbach, Switzerland; 3 Department of Pharmacology, Faculty of Pharmacy, Mahidol University, Bangkok, 10400 Thailand; 4 Faculty of Pharmacy, Center of Biopharmaceutical Science for Healthy Ageing (BSHA), Mahidol University, Bangkok, 10400 Thailand; 5 BioTechMed-Graz, Graz, Austria

**Keywords:** Metabolomics, Molecular medicine

## Abstract

Due to intact reactive oxygen species homeostasis and glucose metabolism, C57BL/6NRj mice are especially suitable to study cellular alterations in metabolism. We applied Nuclear Magnetic resonance spectroscopy to analyze five different tissues of this mouse strain during aging and included female and male mice aged 3, 6, 12, and 24 months. Metabolite signatures allowed separation between the age groups in all tissues, and we identified the most prominently changing metabolites in female and male tissues. A refined analysis of individual metabolite levels during aging revealed an early onset of age-related changes at 6 months, sex-specific differences in the liver, and a biphasic pattern for various metabolites in the brain, heart, liver, and lung. In contrast, a linear decrease of amino acids was apparent in muscle tissues. Based on these results, we assume that age-related metabolic alterations happen at a comparably early aging state and are potentially associated with a metabolic switch. Moreover, identified differences between female and male tissues stress the importance of distinguishing between sexes when studying age-related changes and developing new treatment approaches. Besides, metabolomic features seem to be highly dependent on the genetic background of mouse strains.

## Introduction

During aging, substrate utilization and storage, mitochondrial function, and insulin sensitivity undergo changes that affect cellular metabolism^1,2^. As the global population progressively ages, these age-related metabolic alterations are evident in the increased occurrence of chronic metabolic disorders, including obesity, type 2 diabetes, and cardiovascular diseases^3^. However, specific dietary strategies may be utilized to shape cellular metabolism and, thereby, the aging process^4^. Caloric restriction is, for instance, the most potent intervention to prolong lifespan and improve health in model organisms and mammals^5,6^. Based on these findings, the question regarding the proper timing of such anti-aging strategies arises. Therefore, understanding metabolic signatures at specific ages seems to be essential^7^. A clinical trial investigating 6421 subjects of different ages recently revealed that total energy expenditure peaks in neonates, declines slowly to adult levels at 20 years, and remains stable until 60 years before it drops in old adults. Notably, the relative size and the metabolic activity of specific organs were reported to change during aging^8^, pointing to tissue-specific alterations.

Due to the similarities between mice and humans in tissue aging^9^, mice are frequently used for aging studies. A large cohort study demonstrated that mice reach sexual maturity after 35 days and still continue to grow until 3 months^10^. However, early signs of physical dysfunction were reported already at 6 months^11^. Therefore, mice are defined as mature adults between 3 and 6 months. During this period, which reflects the life phase for humans from 20 to 30 years, mice are fully developed without an < impairment by senescence^10^. Around 9 months, mice already reach perimenopause^12^. Senescent changes in various measures get evident in middle-aged mice between 10 and 14 months, correlating with human age from 38 to 47, and manifest in the whole range of features in mice ranging from 18–24 months, reflecting a period from 56 to 69 years of age in humans. After 24 months, strain-specific diseases are increasingly reported and might mislead the interpretation of aging studies^10^.

Different studies utilized the widely-used C57BL/6 J (B6J) mouse strain^13–15^ to assess metabolic features during aging. However, the B6J strain exhibits a functional deletion in the nicotinamide nucleotide transhydrogenase (*Nnt*) gene^16^. NNT is located in the inner mitochondrial membrane (IMM) and catalyzes the reduction of NADP^+^ to NADPH. Consequently, NNT is essential for detoxifying reactive oxygen species (ROS)^17,18^. In accordance with the function of NNT, the mitochondrial ROS homeostasis was found to be changed in B6J mice, causing enhanced vascular superoxide levels and increased plaque formation in B6J mice than in the C57BL/6 N (B6N) strain^19^. Besides, B6J mice were shown to have a defect in pancreatic beta cell function, causing impaired insulin secretion. A transgenic expression of *Nnt* was found to rescue beta cell function and glucose tolerance, pointing to defective NNT as the leading cause of hampered insulin secretion^20^.

ROS signaling^21^ and glucose metabolism^22^ are both determinants in the process of aging. ROS function as signaling molecules essential to developing antioxidant defense mechanisms boosting health and lifespan. However, overwhelming ROS levels harm DNA, RNA, proteins, and lipids, causing cellular damage and triggering cellular aging^21,23^. In addition, impaired insulin secretion was associated with altered carbohydrate and lipid metabolism^20^. Age-associated insulin resistance might further trigger hyperglycemia-linked age-related diseases like type 2 diabetes mellitus and cardiovascular diseases^22,24,25^. Despite the NNT-linked alterations in ROS homeostasis and insulin-linked metabolism, numerous studies investigated metabolic changes in B6J female and male mice^14,26–28^, while metabolomic patterns during aging in B6N mice are sparse and missed to investigate alterations in females^29^.

Clinical and preclinical aging research has traditionally been male-biased. Due to hormonal fluctuations and the protection of fertile and pregnant women, males were the norm in clinical trials and preclinical experiments. Based on the lack of data regarding female aging, it is still unsolved why women outlive men, although they show worse health at the end of their lives^30^. Sex-chromosomal linked mechanisms and sex-specific hormonal patterns affect metabolism that has been reported to be associated with age-related disorders, like metabolic syndrome, obesity, and diabetes^31^.

Consequently, this study aimed to investigate the metabolomic signatures of female and male B6N mice in different tissues, including the brain, heart, skeletal muscle, liver, and lung, during aging. To cover the aging stages of early adulthood, adulthood without impairment by senescence, middle-age around perimenopause, and old-age before the onset of strain-specific diseases, we analyzed tissues from 3-, 6-, 12-, and 24-months old mice. Our results revealed metabolite clustering at different age stages, sex-specific features, and biphasic patterns of individual metabolites during aging.

## Results

### Age-associated clustering of metabolites in female and male tissues

**Figure 1:**
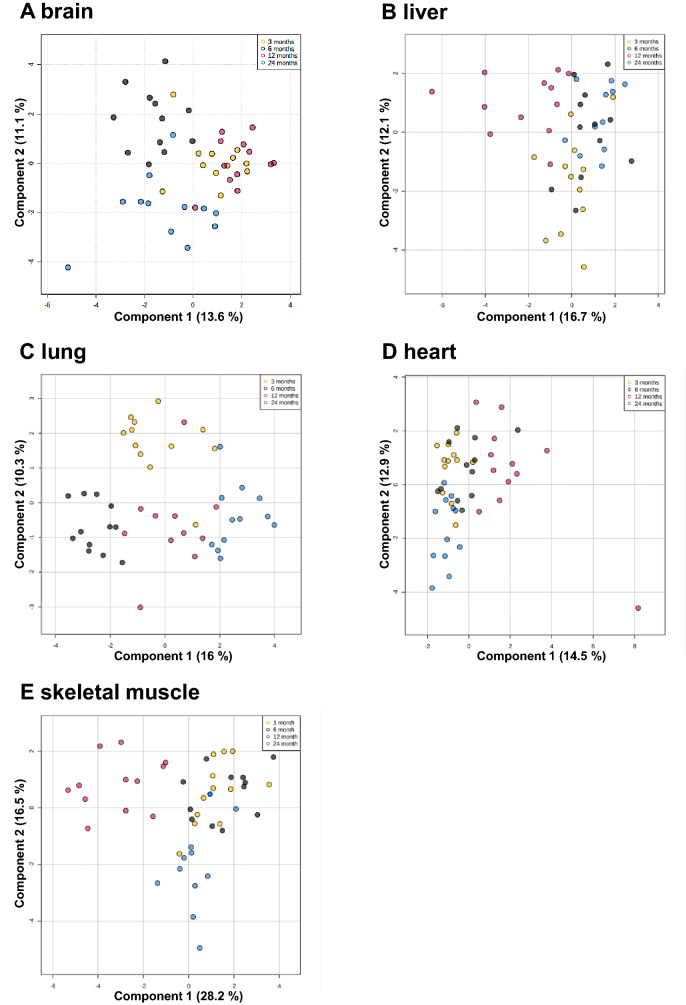
Age group clustering in different murine tissues. S-PLS-DA plot of the brain (**A**), heart (**B**), skeletal muscle (**C**), liver (**D**), and lung (**E**) tissue of six female and six male mice of different ages, including 3 months (1), 6 months (2), 12 months (3), and 24 months (4).

We performed an untargeted metabolomic analysis to determine whether aging correlates with metabolic remodeling in female and male tissues. Therefore, we analyzed the brain, liver, lung, heart, and skeletal muscle tissues of 6 female and 6 male B6N mice at different ages, including 3, 6, 12, and 24 months. Based on results from the NMR analysis, we performed spare and orthogonal partial least squares-discrimination analyses (S-PLS-DA and O-PLS-DA) to determine whether the clustering of metabolites was significantly different between the age groups in various tissues. Given the small sample sizes in the groups, we focused primarily on the qualitative signature, looking for any evidence of the separation between the pre-annotated age groups. SPLS-DA and oPLS-DA (Supplementary Fig. 1) of the respective female and male tissues revealed significant group separation (*p *< 0.05) between distinct age groups for the brain (Fig. 1A), liver (Fig. 1B), lung (Fig. 1C), heart (Fig. 1D), and skeletal muscle (Fig. 1E). The separation between 3 and 12 months in brain tissues was a trend (*p *= 0.08), while the other age groups of brain tissue and all the age groups of the other tissues were significantly clustered (Supplementary Fig. 1). The clustering of the individual female and male age groups for lung tissues was significant (*p *< 0.05). Significant differences between the sexes could be detected for specific age groups in the liver, lung, and heart and for all age groups in skeletal muscle (Supplementary Fig. 1).

### The metabolic signature of brain aging in female and male mice

**Figure 2:**
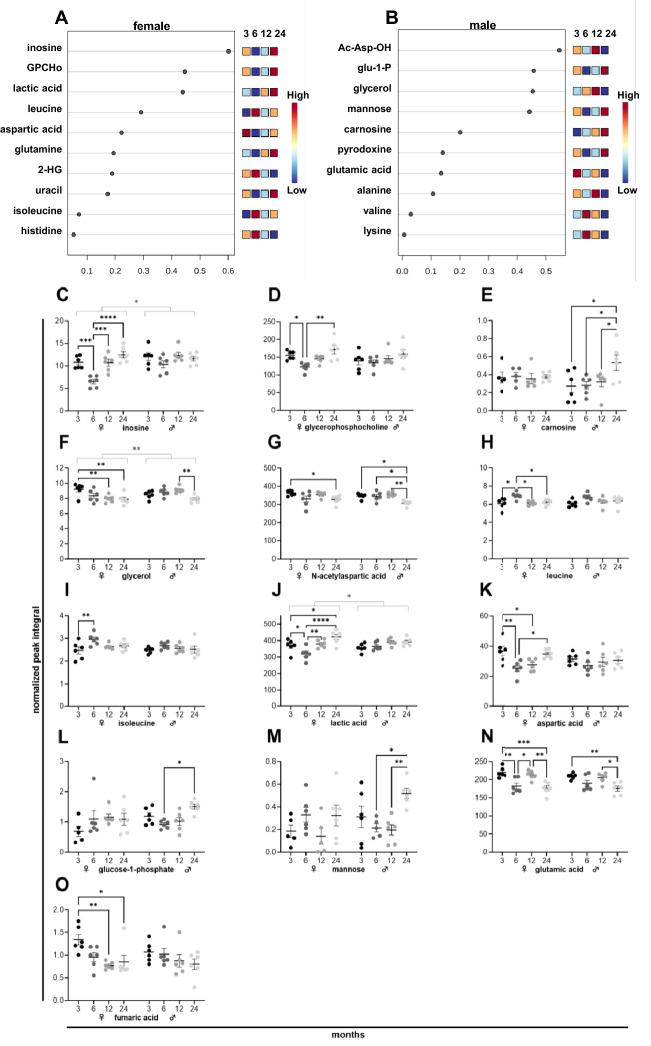
NMR metabolomics of female and male murine brain samples. Metabolites of six female (**A**) and six male (**B**) mice ranked based on the variable importance during projection (VIP) score as a function of age. Individual metabolite levels of female *(left)* and male *(right)* tissues at 3, 6, 12, and 24 months presented as normalized peak integral (**C**–**O**). *2-HG* 2-hydroxyglutaryl, *Ac-Asp-OH* N-acetylaspartic acid, *Glu-1-P* Glucose-1-phosphate, *GPCho* Glycerophosphocholine.

The number of changing metabolites was the highest in brain tissues and was depicted in a heatmap (Supplementary Fig. 2), presenting the average metabolite levels of female and male brain tissues at 3, 6, 12, and 24 months. As shown in the heatmap, changes in metabolite levels differed between female and male brain tissues. To refine this analysis, we analyzed which metabolites of female and male brain tissues underwent the most prominent alterations during aging. We ranked the 10 metabolites with the most significant changes for females (Fig. 2A) and males (Fig. 2B) based on the variable importance during projection (VIP) score as a function of age. For metabolites with significant changes during aging in female or male tissues, the normalized peak integrals at different age stages were presented as scatter plots (MEAN ± SEM). In female brain tissues, the level of neuroprotective metabolites like inosine (Fig. 2C) and glycerophosphocholine (Fig. 2D) decreased from 3 to 6 months but improved during subsequent aging. The neuroprotective carnosine (Fig. 2E) increased in male tissues during aging. The osmoactive glycerol (Fig. 2F) decreased during aging in female and male tissues, and N-acetylaspartic acid (Fig. 2G) dropped at 24 months in male tissues. The levels of BCAAs, including leucine (Fig. 2H) and isoleucine (Fig. 2I), peaked at 6 months in female tissues. Levels of lactic acid (Fig. 2J) and aspartic acid (Fig. 2K) were lowest at 6 months and increased significantly afterward in female tissues. Glucose-1-phosphate (Fig. 2L) and mannose (Fig. 2M) levels were lowest at 6 months and increased at 24 months in male tissues. The levels of glutamic acid (Fig. 2N) dropped in both sexes during aging, and fumaric acid (Fig. 2O) got reduced in female tissues, and a similar trend was detected in male tissues.

### The metabolic signature of liver aging in female and male mice

**Figure 3:**
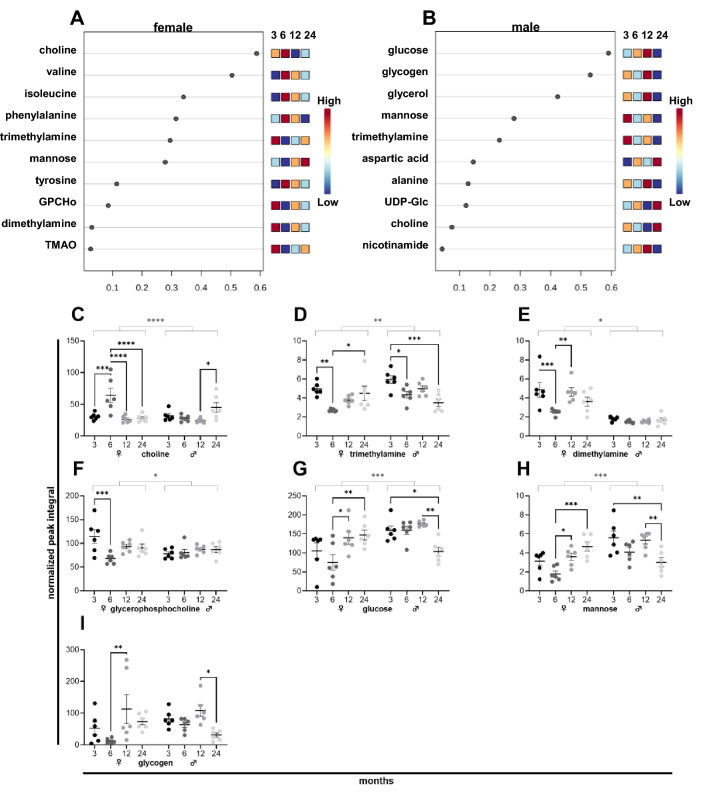
NMR metabolomics of female and male murine liver samples. Metabolites of six female (**A**) and six male (**B**) mice ranked based on the variable importance during projection (VIP) score as a function of age. Individual metabolite levels of female *(left)* and male *(right)* tissues at 3, 6, 12, and 24 months presented as normalized peak integral (**C**–**I**). *GPCho* Glycerophosphocholine, *TMAO* Trimethylamine N-oxide.

Metabolites of female (Fig. 3A) and male (Fig. 3B) liver tissues that underwent the most notable changes during aging are shown in the VIP score. A complete list of changing metabolites in the liver is provided in a heatmap (Supplementary Fig. 3). Liver metabolites that underwent significant changes during aging mainly derived from the glycerophospholipid and glucose metabolism. Thereby, choline (Fig. 3C) peaked at 6 months in female mice. The levels of the choline metabolites trimethylamine (Fig. 3D), dimethylamine (Fig. 3E), and glycerophosphocholine (Fig. 3F) dropped at 6 months, with trimethylamine and dimethylamine increasing during subsequent aging in female tissues. In male liver samples, choline increased at 24 months (Fig. 3C), and trimethylamine got decreased during aging (Fig. 3D). Glucose (Fig. 3G), mannose (Fig. 3H), and glycogen (Fig. 3I) levels were the lowest at 6 months old female mice and reached levels of 3 months old mice during subsequent aging. In contrast, a significant reduction of these metabolites was detected at 24 months in males.

### The metabolic signature of lung aging in female and male mice

**Figure 4:**
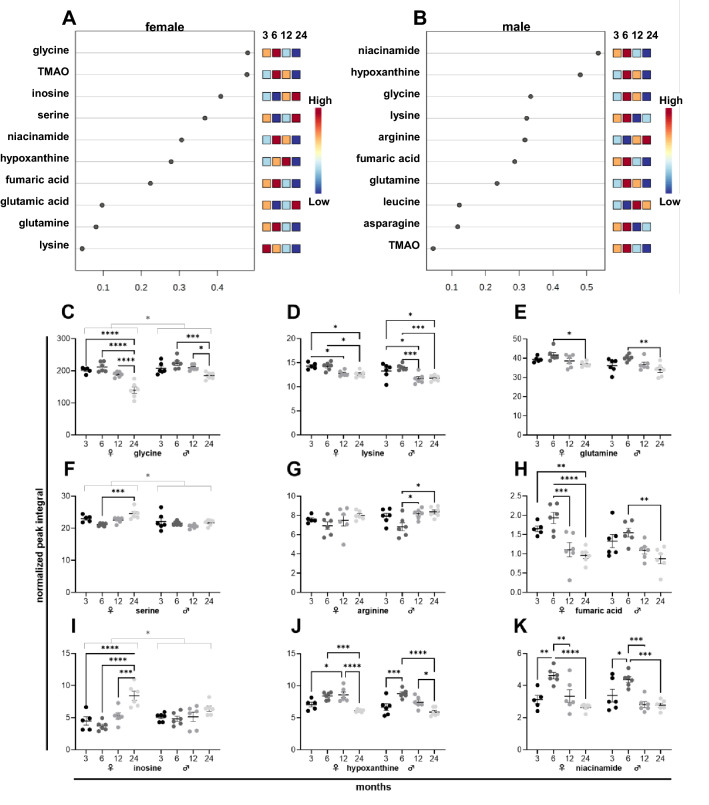
NMR metabolomics of female and male murine lung samples. Metabolites of five female (**A**) and six male (**B**) mice ranked based on the variable importance during projection (VIP) score as a function of age. Individual metabolite levels of female *(left)* and male *(right)* tissues at 3, 6, 12, and 24 months presented as normalized peak integral (**C**–**K**). *TMAO* Trimethylamine N-oxide.

The metabolites changing the most during the process of aging were highly similar between female (Fig. 4A) and male (Fig. 4B) lung tissues. A whole list is provided in a heatmap in Supplementary Fig. 4. The amino acids glycine (Fig. 4C), lysine (Fig. 4D), and glutamine (Fig. 4E) dropped during aging in female and male tissues. Levels of serine (Fig. 4F) and arginine (Fig. 4G) were lowest in female and male tissues at 6 months, respectively. Fumaric acid (Fig. 4H) levels decreased in male and female tissues during aging. Remarkably, levels of inosine (Fig. 4I) significantly increased at 24 months in female tissues, and a similar trend was detected in male tissues. Hypoxanthine (Fig. 4J) and niacinamide (Fig. 4K) levels increased at 6 months and dropped subsequently in both sexes.

### The metabolic signature of heart aging in female and male mice

**Figure 5:**
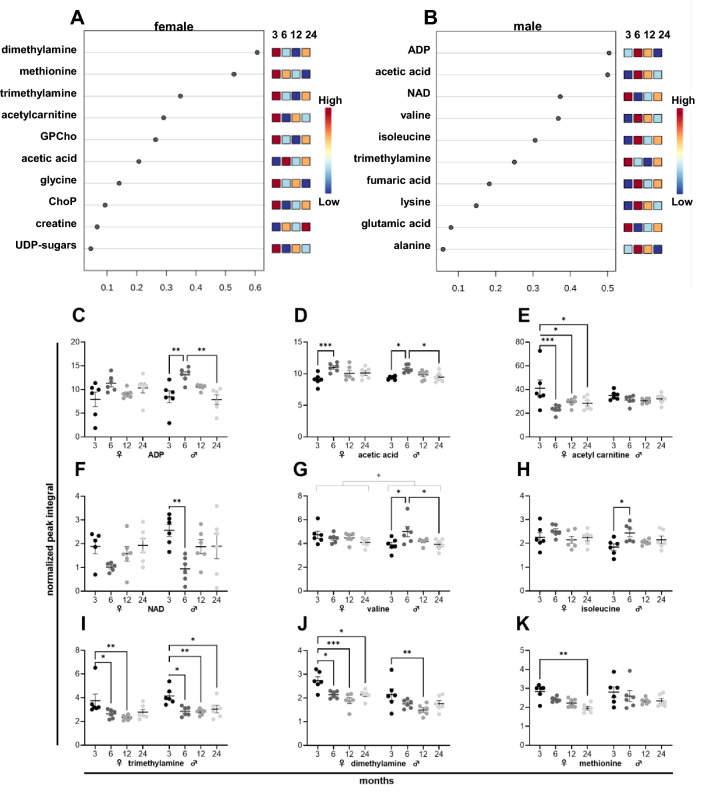
NMR metabolomics of female and male murine heart samples. Metabolites of six female (**A**) and six male (**B**) mice ranked based on the variable importance during projection (VIP) score as a function of age. Individual metabolite levels of female *(left)* and male *(right)* tissues at 3, 6, 12, and 24 months presented as normalized peak integral (**C**–**K**). *ADP* Adenosine diphosphate, *ChoP* Phosphorylcholine, *GPCho* Glycerophosphocholine, *NAD* Nicotinamide adenine dinucleotide.

The changing metabolites of female and male heart tissues over time are depicted in a heatmap (Supplementary Fig. 5). Metabolites of female (Fig. 5A) and male (Fig. 5B) tissues that underwent the most prominent changes during aging are shown in the VIP score. During aging, female and male heart tissues underwent similar but differently pronounced changes in metabolite levels. Levels of adenosine diphosphate (ADP) (Fig. 5C) did not significantly change in female tissues but increased in males. Acetic acid (Fig. 5D) peaked in both sexes at 6 months. In female heart tissues, acetylcarnitine (Fig. 5E) dropped at 6 months. Nicotinamide adenine dinucleotide (NAD) (Fig. 5F) levels were reduced at 6 months in male tissues. Branched-chain amino acids (BCAAs) like valine (Fig. 5G) and isoleucine (Fig. 5H) peaked at 6 months in male tissues. In both sexes, an age-related decrease was detected for trimethylamine (Fig. 5I) and dimethylamine (Fig. 5J). Levels of methionine (Fig. 5K) decreased significantly in female hearts.

### The metabolic signature of skeletal muscle aging in female and male mice

**Figure 6:**
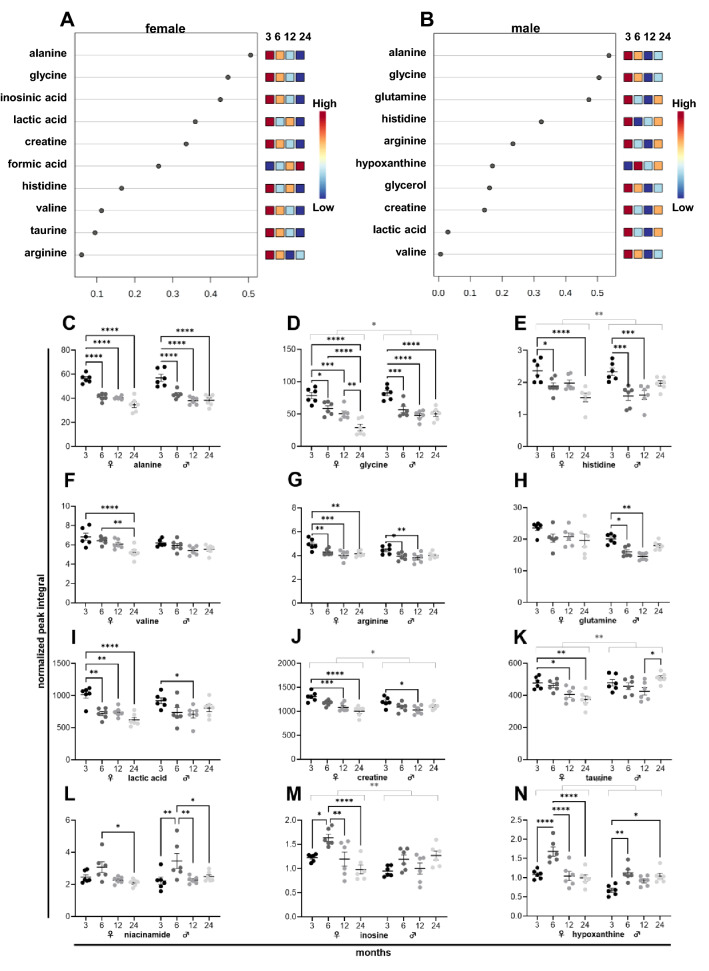
NMR metabolomics of female and male murine skeletal muscle samples. Metabolites of six female (**A**) and six male (**B**) mice ranked based on the variable importance during projection (VIP) score as a function of age. Individual metabolite levels of female *(left)* and male *(right)* tissues at 3, 6, 12, and 24 months presented as normalized peak integral (**C**–**N**).

As in lung tissues, there was a high overlap of metabolites from female (Fig. 6A) and male (Fig. 6B) skeletal muscle tissues in VIP scores. A full list of changing metabolites in skeletal muscle is provided in a heatmap (Supplementary Fig. 6). Aging in skeletal muscle was characterized by a decline in alanine (Fig. 6C), glycine (Fig. 6D), histidine (Fig. 6E), and arginine in both sexes. Valine (Fig. 6F) decreased in females, and glutamine (Fig. 6H) in males. A similar trend was seen in the opposite sex. Besides, lactic acid levels (Fig. 6I) declined during aging in female tissues, and creatinine (Fig. 6J) dropped in both sexes. A difference between male and female skeletal muscle tissues was the reduced level of taurine (Fig. 6K) in aged female tissues. In male tissues, taurine levels were increased in 24 months old mice. The nucleosides niacinamide (Fig. 6L), inosine (Fig. 6M), and hypoxanthine (Fig. 6N) peaked at 6 in female tissues. These alterations were less pronounced in male tissues.

## Discussion

It is central to identify the onset of the aging process to develop efficient strategies for counteracting age-related deterioration. Other reports have already revealed metabolic alterations during aging in B6N mice but focused on a later aging state, defined a broader age range for the individual aging states, and exclusively analyzed male mice^29^. Our study revealed substantial metabolic alterations in the brain, heart, skeletal muscle, liver, and lung of female and male B6N mice at 6 months of age, pointing to the early onset of age-related alterations.

In the brain, similarities in the age-related pattern of individual metabolite levels in female and male tissues were detectable. In female brains, two neuroprotective metabolites, inosine (Fig. 2C) and glycerophosphocholine (Fig. 2D), were reduced from 3 to 6 months and increased during aging. The nucleoside inosine was already previously shown in animal experiments to counteract oxidative alterations in the brain and improve brain function after stroke^32,33^. Glycerophosphocholine normalizes brain choline and acetylcholine levels and membrane fluidity, facilitating learning, memory, and cognitive improvement for patients suffering from, for instance, Alzheimer’s disease^34^. In male brain tissues, carnosine (Fig. 2E) increased steadily during aging and likely conveyed neuroprotection by antioxidative actions, pH-buffering, and anti-glycating activity^35^. Although counteracting mechanisms are in place, brain function deteriorates during aging. We found that the osmoactive glycerol (Fig. 2F) that facilitates brain perfusion^36^ dropped during aging in brain tissues of both sexes, hinting at impaired brain function during aging. That was emphasized by decreased levels of N-acetylaspartic (Fig. 2G) acid in male tissues, a trend also seen in female tissues. That fits reports that identified N-acetylaspartic acid as a marker of brain integrity that gets reduced during aging in humans^37^. Alterations in BCAAs (Fig. 2H,I) as well as in metabolites of carbohydrate metabolism (Fig. 2J-M) and the TCA cycle (Fig. 2N,O) might point to a hampered TCA cycle and impaired mitochondrial metabolism during brain aging.

In liver tissues, the changes in choline and its metabolites and in glucose, mannose, and glycogen, occurred in females and male mice at different aging stages. This might be potentially due to sex-specific differences in hormones during aging. Estrogen levels in females and testosterone levels in males peak around the age of 30 years, correlating with a murine age of 6 months, and decline subsequently during aging^38^. Estrogen substantially affects metabolism. In line with that, it has been reported to improve insulin sensitivity and suppress gluconeogenesis in premenopausal women^39^. Moreover, estrogen is associated with enhanced lipoprotein turnover, potentially increasing the availability of cholesterol and fatty acids for steroidogenesis and the fetal development^40^. In accordance with our findings, decreased glucose, mannose, and glycogen levels in the liver of 6 months female mice might be a potential consequence of estrogen-induced enhancement of carbohydrate metabolism in muscle or adipose tissues. Besides, alterations in choline and its metabolites in 6 months old female mice might be caused by estrogen-induced alterations in lipoprotein formation, including enhanced VLDL formation^41^. During aging, estrogen levels were reduced in females and even exceeded by males^42^. A combination of lower testosterone and stable estrogen levels in old male mice^43^ might induce similar changes in carbohydrate metabolism and lipoprotein turnover as in females at 6 months. However, decreased testosterone levels might be associated with potentially harmful lipoprotein homeostasis with increased LDL and reduced HDL fraction, leading to atherosclerotic lesions^44^.

In the lung and heart tissues, alteration patterns in male and female tissues are largely similar and include biphasic metabolite changes with peak levels of specific amino acids and TCA cycle intermediates at 6 months. Several studies have reported a biphasic pattern in mitochondrial metabolism during aging, including an increased mitochondrial activity during middle age and a subsequent decline during subsequent aging^45^. A biphasic pattern of metabolic parameters, such as mitochondrial activity, oxygen consumption, and acetyl-CoA, was found in various organisms, including *Drosophila melanogaster*^46^, *Caenorhabditis elegans*^47^, *Saccharomyces cerevisiae*^48^, and mice^49^, during aging. The increased mitochondrial metabolic activity might represent a cellular adaptation to the first occurrence of age-associated cellular damages. Age-associated dysfunction of cellular processes including protein folding might require higher levels of mitochondrial ATP production to maintain physiological function. The mitochondrial protein machinery yielding ATP might be deteriorated, and, consequently, a compensatory increase of metabolic intermediates might help to boost mitochondrial metabolic activity to achieve comparable ATP levels during the transition from young adult to middle age. However, dysfunctional mitochondria might potentially produce harmful side-products like reactive oxygen species when firing the electron transport chain to generate ATP^23^. Therefore, reducing mitochondrial metabolic activity and switching to cytosolic ATP production might be necessary. Indeed, glycolysis rates were found to be increased during aging in lung tissues^50^ as well as in aging hearts^51^. This age-related metabolic switch might be partly facilitated by changing sex hormone levels since estrogen and testosterone stimulate mitochondrial biogenesis^52,53^. With respectively peaking estrogen and testosterone levels in females and males between 25 and 30 years^38^, mitochondrial biogenesis might be stimulated until 25–30 years and reduced afterward. Based on these reports, we assume that at 6 months mitochondria are still in full charge of maintaining proper cellular ATP levels. Still, early age-related deterioration might impair their function, which leads to an increase in metabolic intermediates of mitochondrial metabolism, including specific amino acids (Fig. 4C, Fig. 4D,E) and fumaric acid (Fig. 4H) in the lung as well as amino acids (Fig. 5G, Fig. 5H), acetic acid (Fig. 5D), and ADP (Fig. 5C) in the heart. These increased levels of intermediates might help to stimulate mitochondria, but might also simply reflect an accumulation due to mitochondrial dysfunction. In any case, a metabolic switch with increased rates of cytosolic ATP production necessary might.

Besides, in the female and male lung tissues, alterations in metabolites of purine metabolism, associated with lung inflammation^54^, were found. Given the changes in hypoxanthine (Fig. 4J) and niacinamide (Fig. 4K) levels at 6 months, substantial changes in the inflammatory response of lung tissues initiated during middle age might be caused by to mitochondrial dysfunction associated with ROS production^55^. In male and female heart tissues, trimethylamine (Fig. 5I), dimethylamine (Fig. 5J), and methionine (Fig. 5K) levels decreased during aging, pointing to changes in choline metabolism^56^ and potentially alterations in methylation levels in heart tissues since methionine functions as a methyl donor^57^.

In skeletal muscle, a substantial alteration in amino acid levels is the key feature of aging in male and female tissues. The levels of several amino acids, including alanine (Fig. 6C), glycine (Fig. 6D), histidine (Fig. 6E), valine (Fig. 6F), arginine (Fig. 6G), and glutamine (Fig. 6H), were steadily decreasing in female and male skeletal muscle tissues during aging. Reduced levels of amino acids might be due to impaired amino acid uptake^58^ or resulting from enhanced amino acid metabolism^59^ with aging. The level of lactic acid (Fig. 6I) decreased during aging in muscle tissues in female tissues. This finding is well-aligned with reports about aging rats^60^ and might be related to a reduced lactate dehydrogenase activity as reported for aged humans^61^. In addition, levels of creatinine (Fig. 6J) also steadily declined during aging in muscle tissues of female and male mice. These results suggest that anaerobic metabolism is hampered during aging and fit the reported reduction in anaerobic work capacity in aged athletes^62^. A shift in metabolism towards amino acid utilization might also affect decreased protein synthesis related to age-associated loss in muscle mass and strength reported in humans^63^. Notably, the level of taurine (Fig. 6K) was solely reduced in female muscle tissues. This might be a potential reason for the reported increased sarcopenia rates in females under 70 years^64^ and the higher potential for men to prevent sarcopenia by physical activity^65^. Enhanced levels of niacinamide (Fig. 6L), inosine (Fig. 6M), and hypoxanthine (Fig. 6N) at 6 months point to changes in the purine metabolism and potentially to a restructuring of muscle fibers^66–68^ in females at an early stage of aging.

We assume that B6N mice might be a valuable model to study age-related metabolic alterations while aging in B6J might be affected by defective NNT. In an analysis of liver and muscle tissues of 3 and 22 months old B6J mice by GC/MS and LC-MS/MS, an accumulation of glycogen intermediates in both tissues pointed to altered glycogen metabolism. Besides, increased lactate levels and a reduction in glycolytic intermediates suggested elevated anaerobic glycolysis^15^. In contrast, our NMR analysis in B6N mice revealed a drop in glucose and glycogen levels in the liver, pointing to a crucial metabolic difference between B6J and B6N mice. However, for a direct comparison between B6J and B6N strains, levels of intermediates of the glucose and fatty acid metabolism should be obtained by the same method.

In a previous study, we analyzed metabolomic changes in the brain, heart, liver, kidney, lung, and spleen tissues of mice with the mixed genetic background of 129 J and C57BL/6 during aging^27^. Given that in the current study, we used the same preparation procedure, measurement settings, and data processing techniques, the metabolic discrepancies between female mice with the mixed genetic background of 129 J and C57BL/6 compared to B6N mice are striking. Most age-associated alterations between 9–10 week and 96–104 week-old female B6J mice could not be found in the comparison between 3 and 24-months-old B6N mice. Interestingly, in 24-months-old B6J mice and 6-months-old B6N mice, a similar trend in metabolite patterns was detected in comparison to the respective young mice. In the brain of female B6N mice, levels of inosine and lactate dropped while amino acids like leucine and isoleucine peaked at 6 months. All these alterations were subsequently normalized in B6N, while in B6J mice, these changes happened at old age. In heart tissues of female B6N mice, levels of valine and isoleucine, as well as acetic acid, peaked at 6 months. These levels were normalized at old age when amino acids and acetic acid levels increased in B6J mice. In the liver, most of the metabolites changing in B6N mice remained unchanged in B6J mice, except for glucose which dropped in B6N mice at 6 months and in B6J mice at 24 months. In the lung, levels of amino acids increased in aged B6J mice, while glycine, lysine, and glutamine dropped in B6N mice. Notably, in both mouse strains, decreased levels of niacinamide were found at the old age around 24 months^27^. These results might indicate that a metabolic switch happens in distinct mouse strains at different aging periods. Since the B6N mouse strain harbors functional NNT, it seems tempting to speculate that the biphasic mode of mitochondrial aging is due to alterations in ROS homeostasis.

A previous study has also investigated the alterations during aging in B6N mice. Thereby, solely male mice were included and split into young (3–8 months), adult (13–23 months), and old (27–36 months) mice. This study nicely correlated signatures associated with frailty with metabolic changes but did not present levels of individual metabolites during aging. Based on the results presented as VIP scores and correlation coefficients, biphasic patterns of several metabolites with a peak or drop at middle age–defined as a period between 13 and 23 months—were also present in studied tissues, including the brain, heart, liver, skeletal muscle, and serum. Unfortunately, the differently defined aging groups make a comparison to our study impossible. Previous work has revealed the frequent onset of strain-specific diseases after 24 months^10^. Consequently, it seems questionable whether results obtained from an old aging cohort consisting of mice between 27 and 36 mice are indeed reflecting metabolic changes during aging.

Based on the results of different studies investigating metabolic changes during aging, the composition of the individual aging cohorts and the genetic background of the various strains are crucial to the metabolomic alterations during aging. Besides, different diets and housing conditions seem to be significant determinants of the metabolome. In addition, results might be affected to a minor extent by sample preparation and analysis.

To our understanding, B6N are highly suitable for aging studies and comparison with humans since they have a functional NNT. The data hereby revealed significant alterations in the brain, heart, skeletal muscle, liver, and lung samples during aging and identified sex-specific separation of muscle tissues at specific aging stages in B6N mice. Interestingly, age-related metabolic patterns of individual metabolites were comparable between the female and male brain, heart, skeletal muscle, and lung tissues. In contrast, the metabolite signatures of individual metabolites of females and males differed substantially in liver samples during aging, pointing to a potential impact of sex-specific hormones.

Overall, individual metabolite levels point to an early onset of age-related alterations in female and male tissues at already 6 months. Thereby, crucial metabolites changed biphasically with a peak or drop at 6 months, followed by subsequent normalization during aging. These changes might be assumed to reflect a metabolic switch during early aging.

In summary, our data obtained from B6N mice reveal the importance of studying the aging process distinctly in female and male tissues and of including and differentiating cohorts with a comparably early aging state. Furthermore, differences to former studies in B6J mice, exhibiting disturbed ROS homeostasis and carbohydrate metabolism, highlight the need to carefully select mouse strains to gain valuable data from aging studies. Given the search for successful anti-aging strategies, studies like this will help unveil the right timing for intervention and potentially the need for distinct treatment options for females and males.

## Methods

### Murine handling

C67BL/6NRj (B6N) mice (Janvier Labs; Le Genest-Saint-Isle, France) were housed in a temperature-controlled facility with a 12 h light–dark cycle and aged in-house. A standard chow diet (#3437 Granovit AG; Kaiseraugst, Suisse) and water were provided *ad libidum*. Mice of the different aging cohorts, including 3, 6, 12, or 24 months, were sacrificed within 1.5 years by placing them under CO_2_ narcosis for 3 min (CO_2_ flow was 50% of cage volume per minute), followed by cervical dislocation. Whole organs of 6 male and 6 female mice were subsequently shock-frozen in liquid nitrogen and stored at -80 °C. For the brain samples, the hypothalamus was removed before shock-freezing.

### Ethical declaration

The ethics committee of the Veterinary Office (ZH112/19) of Canton Zurich, Switzerland, has approved the study on B6N mice, and all methods were carried out in accordance with relevant guidelines and regulations. The study was carried out in compliance with the ARRIVE guidelines.

### NMR Sample Preparation for metabolomic analysis

Murine samples were snap-frozen in liquid nitrogen and stored at −80 °C until sample preparation. 30–70 mg of respective organs were used for NMR metabolomics analysis. Each tissue had 12 biological replicates. Samples were suspended in 400 µl of ice-cold methanol and 200 µl of MilliQ H_2_O_2_ and transferred to Precellys tubes with 1.4 mm diameter zirconium oxide beads (Bertin Technologies; Villeurbanne, France). This suspension was homogenized two times for 20 s by Precellys24 tissue homogenizer at 25 °C (Bertin Technologies, Montigny-le-Bretonneux, France). Afterward, the homogenized samples were centrifuged at 10,000 rpm for 30 min at 4 °C. The supernatant was transferred to a new tube for metabolomics analysis. Supernatant samples were lyophilized at < 1 Torr, 850 rpm, 25 °C for 10 h in a vacuum-drying chamber (Savant Speedvac SPD210 vacuum concentrator), with an attached cooling trap (Savant RVT450 refrigerated vapor trap) and vacuum pump (VLP120) (Thermo Scientific; Waltham, Ma, USA). Subsequently, samples were re-dissolved in 500 µl of NMR buffer containing 0.08 M Na_2_HPO_4_, 5 mM 3-trimethylsilyl propionic acid-2,2,3,3,-d4 sodium salt (TSP) and 0.04 (w/v)% NaN_3_ in D_2_O, adjusted to 7.4 pH with 8 M HCl and 5 M NaOH^27^.

The prepared metabolic samples were measured at 310 K using a 600 MHz Bruker Avance Neo NMR spectrometer equipped with a TXI 600S3 probe head. The Carr–Purcell– Meiboom–Gill (CPMG) pulse sequence was used to acquire 1H 1D NMR spectra with a pre-saturation for water suppression (cpmgpr1d, 512 scans, 73728 points in F1, 12019.230 Hz spectral width, 1024 transients, recycle delay 4 s)^69,70^.

### NMR data acquisition, analysis, and visualization

NMR spectral data were processed as previously described^27^. The data were processed in Bruker Topspin version 4.0.2 using one-dimensional exponential window multiplication of the FID, Fourier transformation, and phase correction. The NMR data were then imported into Matlab2014b, TSP was used as the internal standard for chemical-shift referencing (set to 0 ppm), regions around the water, TSP and methanol signals were excluded, the NMR spectra were aligned, and a probabilistic quotient normalization was performed. Metabolite identification was carried out using Chenomx NMR Suite 8.4 (Chenomx Inc., Edmonton, AB, Canada) and reference compounds. Quantification of metabolites was carried out by signal integration of normalized spectra. For each metabolite, a representative peak with no overlapping signals was identified, the start and endpoints of the integration were chosen to revolve around that peak, and the area of the peak was integrated by summing up the value for each point. Orthogonal partial least squares discriminant analysis (oPLS-DA) and sparse partial least squares-discriminant analysis (sPLS-DA) were performed in Matlab2014b and MetaboAnalyst 5.0, as well as all associated data consistency checks and cross-validation^71^. The statistical significance of the determined differences was validated by the quality assessment statistic Q2 and presented as specific p-values. For visualization of the metabolite data, heatmaps and variable importance during projection (VIP) scores were made with MetaboAnalyst 5.0. For metabolites with significant changes during aging in female or male tissues, the normalized peak integrals at different age stages were presented as scatter plots (MEAN ± SEM).

GraphPad Prism 9.3.1 (GraphPad Software, La Jolla, CA). Two- way ANOVA with Tukey’s multi-comparison test was performed to assess statistical differences that were presented as specific p-values (* < 0.05, ** < 0.01, *** < 0.001).

## Supplementary Information

Supplementary Information 1.

Supplementary Information 2.

## Data Availability

The dataset used and analyzed during the current study is available in Supplementary Dataset 1.
