# Supplementary material for: The sex-specific metabolic signature of C57BL/6NRj mice during aging
Source: Sci Rep. 2022 Dec 6;12:21050. doi: 10.1038/s41598-022-25396-8 (PMC9726821; doi:10.1038/s41598-022-25396-8)
Supplement: Supplementary file 1 — Supplementary Information 1. [file 41598_2022_25396_MOESM1_ESM.pdf]

The sex-specific metabolic signature of C57BL/6NRj mice during aging

Doruntina Bresilla[1], Hansjoerg Habisch[1], Iva Pritišanac[1], Kim Zarse[2], Warisara Parichatikanond[3,4], Michael Ristow[2], Tobias Madl[1,5,*], and Corina T. Madreiter-Sokolowski[1,5,*]

[1]Molecular Biology and Biochemistry, Gottfried Schatz Research Center, Medical University of Graz, Neue Stiftingtalstraße 6/VI, 8010 Graz, Austria

[2]Laboratory of Energy Metabolism, Institute of Translational Medicine, Department of Health Sciences and Technology, ETH Zurich, Schorenstrasse 16, 8603 Schwerzenbach, Switzerland

[3]Department of Pharmacology, Faculty of Pharmacy, Mahidol University, 10400 Bangkok, Thailand

[4]Center of Biopharmaceutical Science for Healthy Ageing (BSHA), Faculty of Pharmacy, Mahidol University, 10400 Bangkok, Thailand

[5]BioTechMed-Graz, Graz, Austria

[*]Correspondence should be addressed to: corina.madreiter@medunigraz.at, tobias.madl@medunigraz.at

Supplementary Fig. 1

Brain

MIXED

Q^2	3 m	6 m	12 m	24 m
3 m		0.40	0.28	0.85
6 m			0.54	0.60
12 m				0.67
24 m				

p	3 m	6 m	12 m	24 m
3 m		0.01	0.08	<0.01
6 m			0.01	<0.01
12 m				<0.01
24 m				

FEMALE

Q^2	3 m	6 m	12 m	24 m
3 m		0.61	0.69	0.78
6 m			0.84	0.81
12 m				0.84
24 m				

p	3 m	6 m	12 m	24 m
3 m		0.03	0.04	<0,01
6 m			<0,01	<0,01
12 m				<0,01
24 m				

MALE

Q^2	3 m	6 m	12 m	24 m
3 m		0.47	0.14	0.72
6 m			0.34	0.68
12 m				0.79
24 m				

p	3 m	6 m	12 m	24 m
3 m		0.11	0.27	0.01
6 m			0.16	0.01
12 m				0.01
24 m				

FEMALE VS MALE

Q^2	3 m, m	6 m, m	12 m, m	24 m, m
3 m, f	-0.90			
6 m, f		0.47		
12 m, f			0.17	
24 m, f				0.50

p	3 m, m	6 m, m	12 m, m	24 m, m
3 m, f	0.57			
6 m, f		0.15		
12 m, f			0.41	
24 m, f				0.06

Liver

MIXED

Q^2	3 m	6 m	12 m	24 m
3 m		0.65	0.75	0.41
6 m			0.54	0.87
12 m				0.38
24 m				

p	3 m	6 m	12 m	24 m
3 m		<0.01	<0.01	<0.01
6 m			<0.01	<0,01
12 m				0.02
24 m				

FEMALE

Q^2	3 m	6 m	12 m	24 m
3 m		0.59	0.74	0.79
6 m			0.56	0.61
12 m				0.20
24 m				

p	3 m	6 m	12 m	24 m
3 m		0.02	0.01	0.01
6 m			0.02	0.01
12 m				0.45
24 m				

MALE

Q^2	3 m	6 m	12 m	24 m
3 m		0.88	0.73	0.56
6 m			0.76	0.80
12 m				0.75
24 m				

p	3 m	6 m	12 m	24 m
3 m		<0.01	0.04	0.06
6 m			0.01	<0.01
12 m				<0.01
24 m				

FEMALE VS MALE

Q^2	3 m, m	6 m, m	12 m, m	24 m, m
3 m, f	0.79			
6 m, f		0.81		
12 m, f			0.81	
24 m, f				0.16

p	3 m, m	6 m, m	12 m, m	24 m, m
3 m, f	<0.01			
6 m, f		<0.01		
12 m, f			<0.01	
24 m, f				0.28

Lung

MIXED

Q^2	3 m	6 m	12 m	24 m
3 m		0.78	0.56	0.70
6 m			0.82	0.90
12 m				0.82
24 m				

p	3 m	6 m	12 m	24 m
3 m		<0.01	<0.01	<0.01
6 m			<0.01	<0.01
12 m				<0.01
24 m				

FEMALE

Q^2	3 m	6 m	12 m	24 m
3 m		0.90	0.81	0.90
6 m			0.87	0.93
12 m				0.78
24 m				

p	3 m	6 m	12 m	24 m
3 m		0.01	<0.01	<0.01
6 m			<0.01	<0.01
12 m				0.01
24 m				

MALE

Q^2	3 m	6 m	12 m	24 m
3 m		0.60	0.79	0.72
6 m			0.91	0.95
12 m				0.84
24 m				

p	3 m	6 m	12 m	24 m
3 m		0.03	0.03	<0.01
6 m			<0.01	0.01
12 m				<0.01
24 m				

FEMALE VS MALE

Q^2	3 m, m	6 m, m	12 m, m	24 m, m
3 m, f	0.75			
6 m, f		-0.70		
12 m, f			0.77	
24 m, f				0.77

p	3 m, m	6 m, m	12 m, m	24 m, m
3 m, f	0.01			
6 m, f		0.87		
12 m, f			<0.01	
24 m, f				<0.01

Heart

MIXED

Q^2	3 m	6 m	12 m	24 m
3 m		0.75	0.77	0.37
6 m			0.66	0.71
12 m				0.45
24 m				

p	3 m	6 m	12 m	24 m
3 m		<0.01	<0.01	0.01
6 m			<0.01	<0.01
12 m				<0.01
24 m				

FEMALE

Q^2	3 m	6 m	12 m	24 m
3 m		0.57	0.54	0.73
6 m			0.54	0.71
12 m				0.64
24 m				

p	3 m	6 m	12 m	24 m
3 m		0.01	0.13	0.03
6 m			0.1	0.03
12 m				0.03
24 m				

MALE

Q^2	3 m	6 m	12 m	24 m
3 m		0.78	0.69	0.14
6 m			0.81	0.68
12 m				0.52
24 m				

p	3 m	6 m	12 m	24 m
3 m		<0.01	0.04	0.42
6 m			0.01	<0.01
12 m				0.05
24 m				

FEMALE VS MALE

Q^2	3 m, m	6 m, m	12 m, m	24 m, m
3 m, f	0.63			
6 m, f		0.58		
12 m, f			0.49	
24 m, f				0.09

p	3 m, m	6 m, m	12 m, m	24 m, m
3 m, f	0.18			
6 m, f		0.02		
12 m, f			0.03	
24 m, f				0.33

Skeletal muscle

MIXED

Q^2	3 m	6 m	12 m	24 m
3 m		0.81	0.77	0.82
6 m			0.28	0.62
12 m				0.45
24 m				

p	3 m	6 m	12 m	24 m
3 m		<0.01	<0.01	<0.01
6 m			0.02	<0.01
12 m				<0.01
24 m				

FEMALE

Q^2	3 m	6 m	12 m	24 m
3 m		0.81	0.81	0.87
6 m			0.22	0.85
12 m				0.47
24 m				

p	3 m	6 m	12 m	24 m
3 m		<0.01	<0.01	<0.01
6 m			0,15	0.01
12 m				0.05
24 m				

MALE

Q^2	3 m	6 m	12 m	24 m
3 m		0.73	0.93	0.72
6 m			0.65	0.56
12 m				0.66
24 m				

p	3 m	6 m	12 m	24 m
3 m		<0.01	<0.01	<0.01
6 m			0.06	0.04
12 m				0.01
24 m				

FEMALE VS MALE

Q^2	3 m, m	6 m, m	12 m, m	24 m, m
3 m, f	0.85			
6 m, f		0.73		
12 m, f			0.94	
24 m, f				0.85

p	3 m, m	6 m, m	12 m, m	24 m, m
3 m, f	<0.01			
6 m, f		<0.01		
12 m, f			<0.01	
24 m, f				<0.01

Supplementary Figure 1. sPLS analyses showing differences between age groups consisting of females and males (mixed), between age groups including females or males exclusively, as well as between the sexes at a specific age (female vs. male). Six female and six male mice were included.

Supplementary Fig. 2

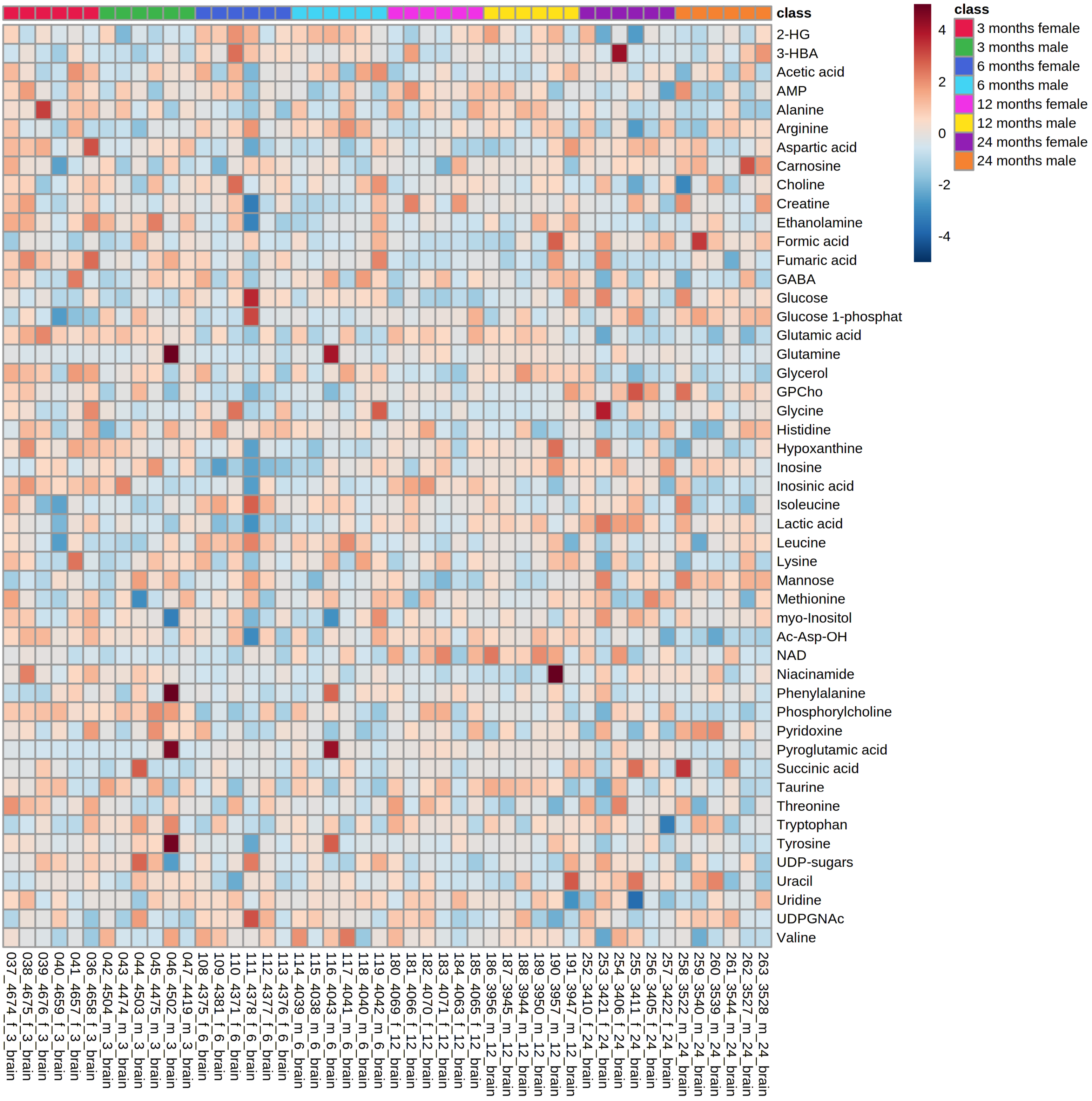

Supplementary Figure 2. Heatmap of NMR analysis showing the relative metabolite levels (mean) in the brain from six female and six male tissues. 1 = female, 3 months; 2 = male, 3 months; 3 = female, 6 months; 4 = male, 6 months; 5 = female, 12 months; 6 = male, 12 months, 7 = female, 24 months; 8 = male, 24 months. Abbreviations: 2-HG: 2-hydroxyglutaryl, 3-HBA: 3-hydroxybutyric acid, Ac-Asp-OH: N-acetyaspartic acid, AMP: adenosine monophosphate, GABA: gamma-aminobutyric acid, GPCho: glycerophosphocholine, UDPGNAc: uridine diphosphate-N-acetylglucosamine.

Supplementary Fig. 3

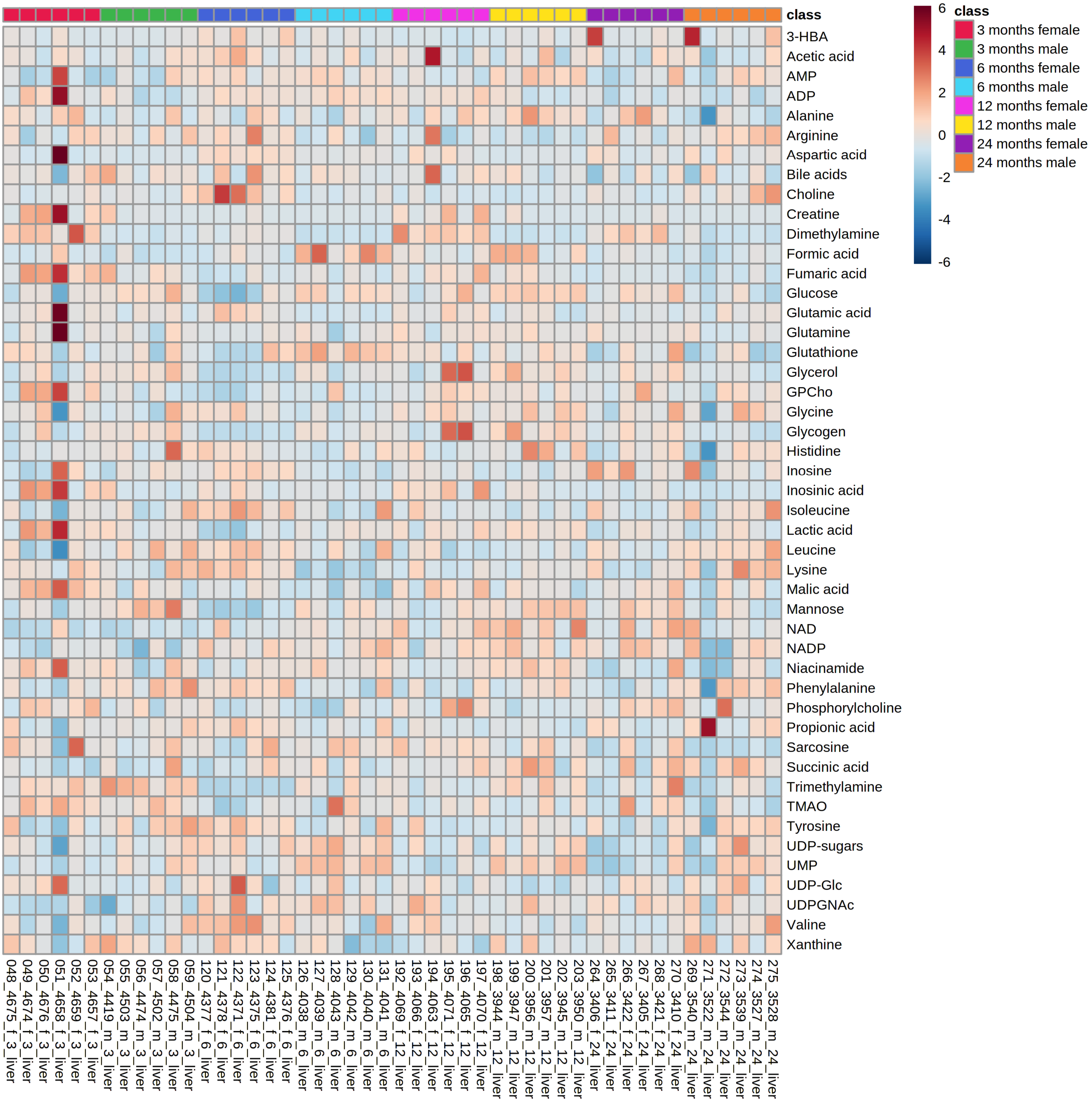

Supplementary Figure 3. Heatmap of NMR analysis showing the relative metabolite levels (mean) in the liver from six female and six male tissues. 1 = female, 3 months; 2 = male, 3 months; 3 = female, 6 months; 4 = male, 6 months; 5 = female, 12 months; 6 = male, 12 months, 7 = female, 24 months; 8 = male, 24 months. Abbreviations: 3-HBA: 3-hydroxybutyric acid, AMP: adenosine monophosphate, ADP: adenosine diphosphate, GPCho: glycerophosphocholine, NAD: nicotinamide adenine dinucleotide, NADP: nicotinamide adenine dinucleotide phosphate), TMAO: trimethylamine N-oxide, UDPGNAc: uridine diphosphate-N-acetylglucosamine, UDP-Glc: uridine diphosphate glucose, UMP: uridine 5'-monophosphate.

Supplementary Fig. 4

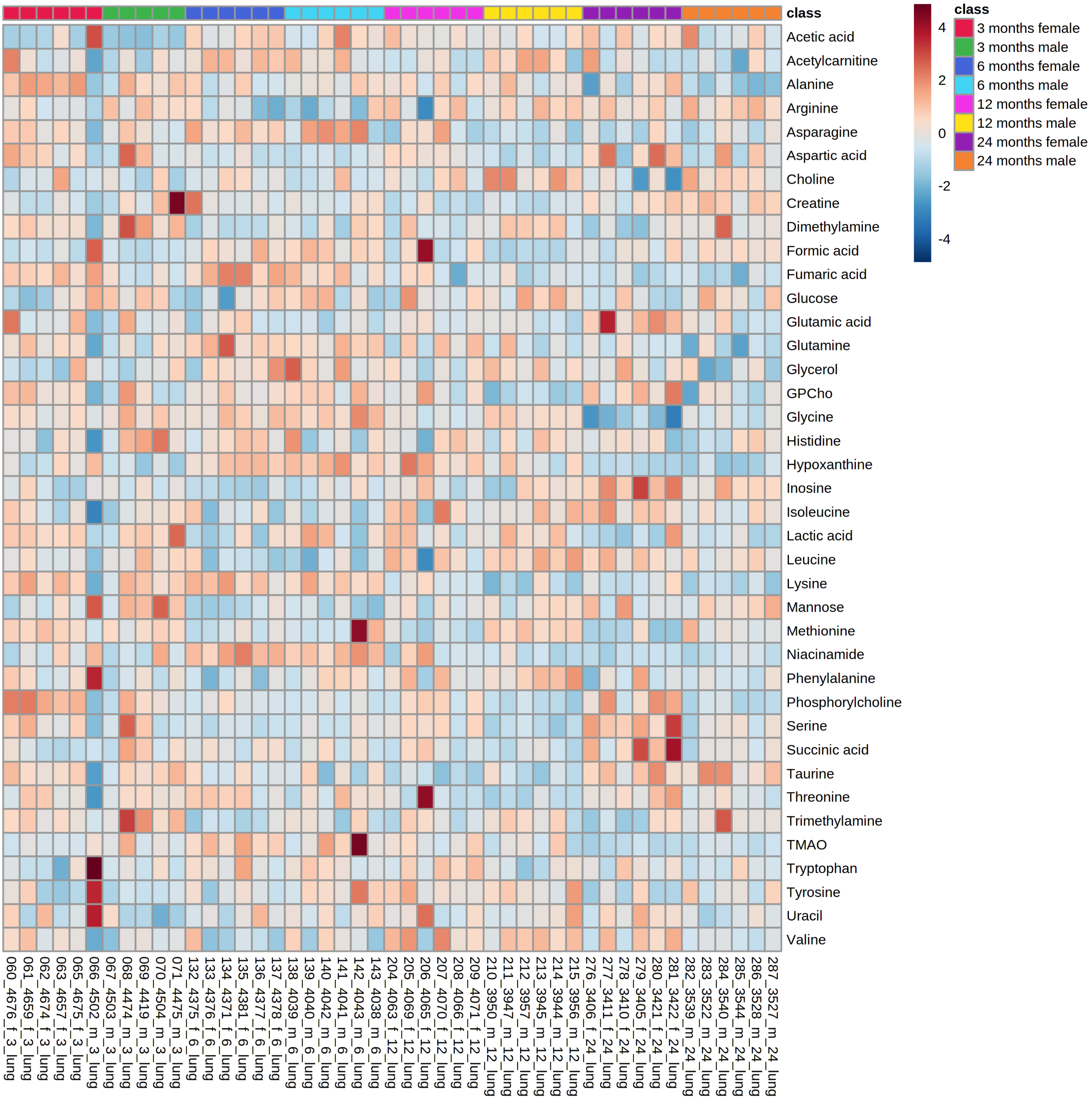

Supplementary Figure 4. Heatmap of NMR analysis showing the relative metabolite levels (mean) in the lung from five female and six male tissues. 1 = female, 3 months; 2 = male, 3 months; 3 = female, 6 months; 4 = male, 6 months; 5 = female, 12 months; 6 = male, 12 months, 7 = female, 24 months; 8 = male, 24 months. Abbreviations: GPCho: glycerophosphocholine, TMAO: trimethylamine N-oxide.

Supplementary Fig. 5

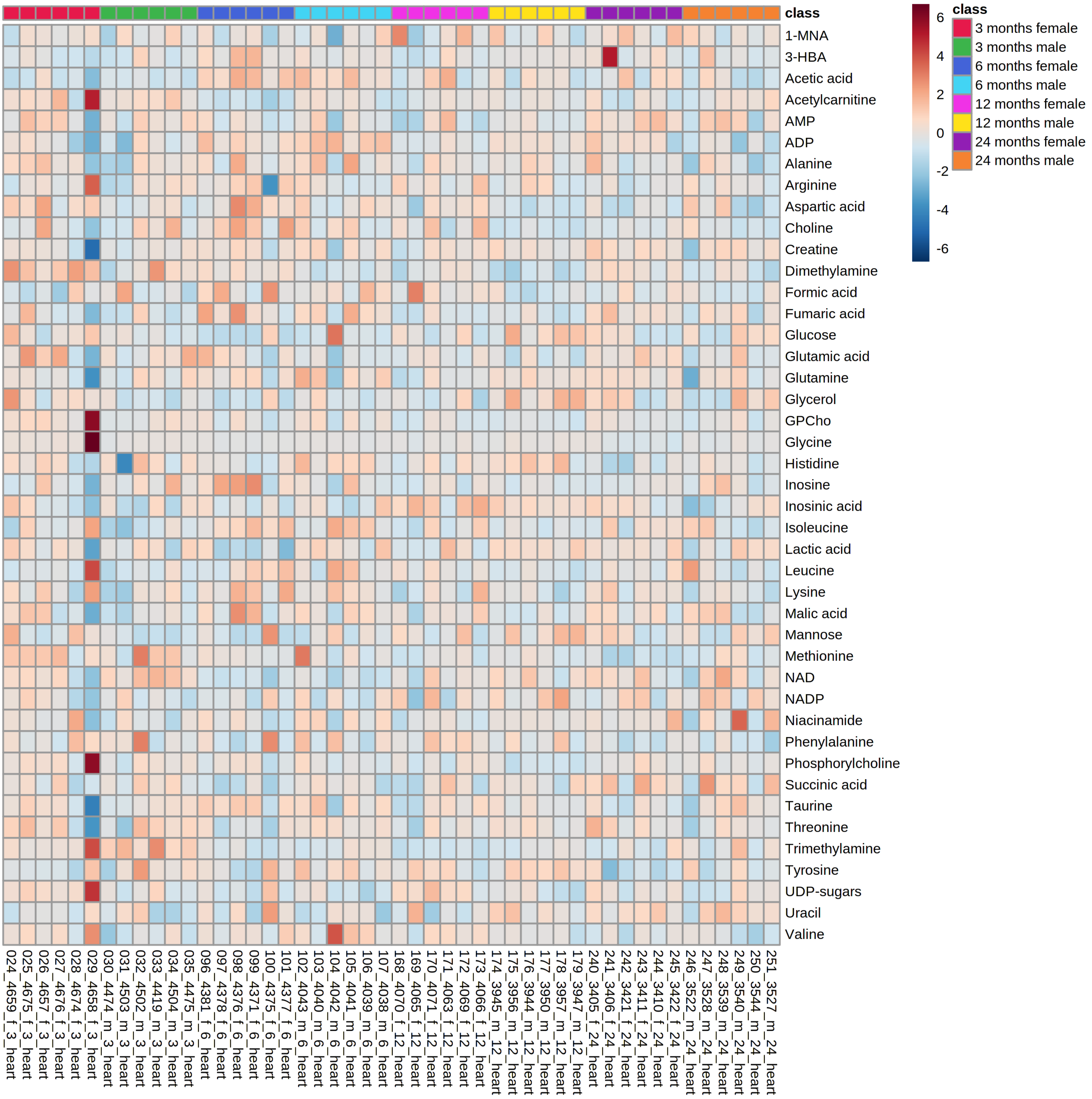

Supplementary Figure 5. Heatmap of NMR analysis showing the relative metabolite levels (mean) in the heart from six female and six male tissues. 1 = female, 3 months; 2 = male, 3 months; 3 = female, 6 months; 4 = male, 6 months; 5 = female, 12 months; 6 = male, 12 months, 7 = female, 24 months; 8 = male, 24 months. Abbreviations:1-MNA: 1-Methylnicotinamide, 3-HBA: 3-hydroxybutyric acid, AMP: adenosine monophosphate, ADP: adenosine diphosphate, GPCho: glycerophosphocholine, NAD: nicotinamide adenine dinucleotide, NADP nicotinamide adenine dinucleotide phosphate.

Supplementary Fig. 6

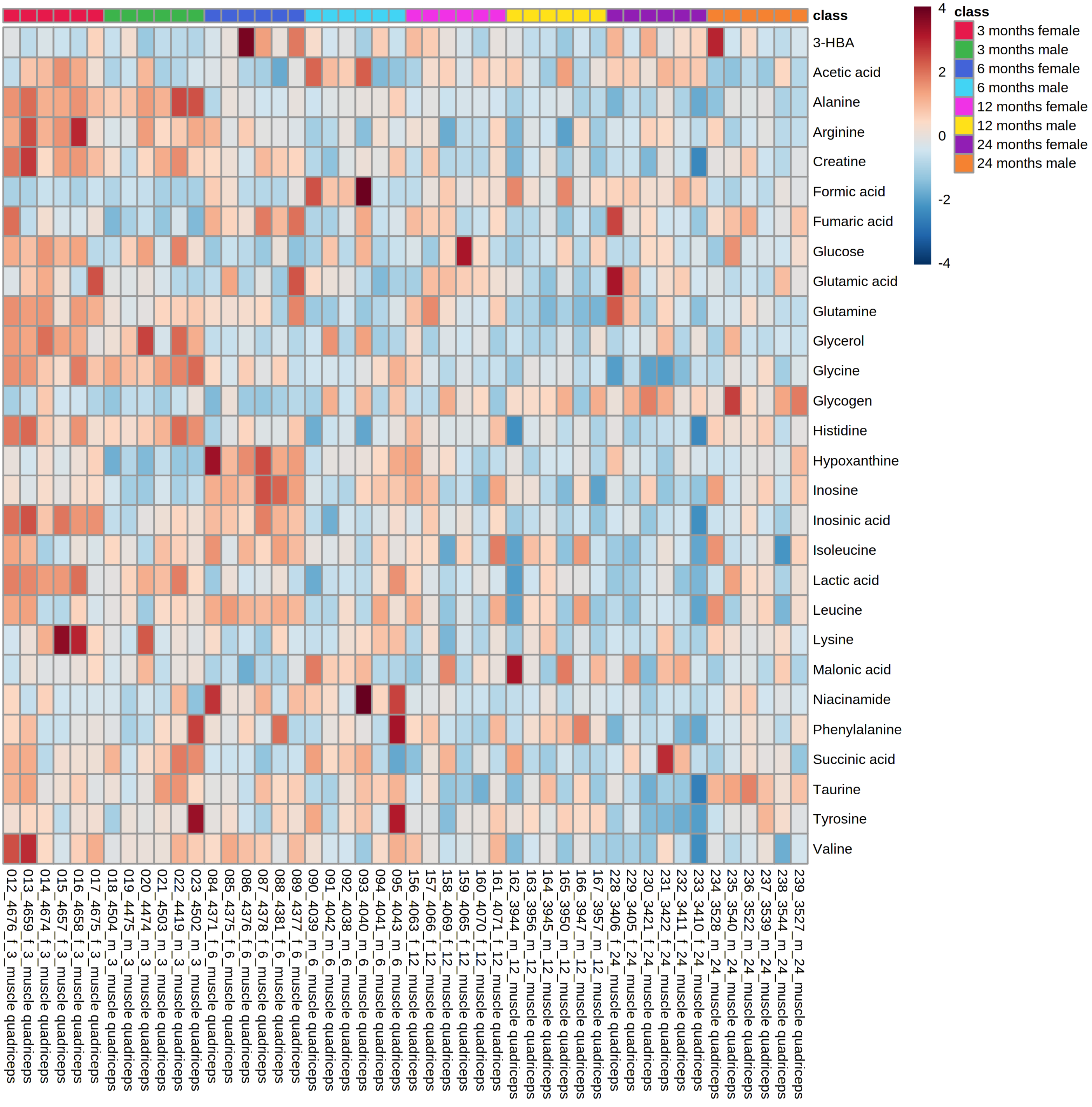

Supplementary Figure 6. Heatmap of NMR analysis showing the relative metabolite levels (mean) in skeletal muscle from six female and six male tissues. 1 = female, 3 months; 2 = male, 3 months; 3 = female, 6 months; 4 = male, 6 months; 5 = female, 12 months; 6 = male, 12 months, 7 = female, 24 months; 8 = male, 24 months. Abbreviations: 3-HBA: 3-hydroxybutyric acid.
